# Task-dependent neural bases of perceiving emotionally expressive targets

**DOI:** 10.3389/fnhum.2012.00228

**Published:** 2012-08-02

**Authors:** Jamil Zaki, Jochen Weber, Kevin Ochsner

**Affiliations:** ^1^Department of Psychology, Harvard UniversityCambridge, MA, USA; ^2^Department of Psychology, Columbia UniversityNew York, NY, USA

**Keywords:** emotional expressivity, empathy, fMRI, medial prefrontal cortex, social cognition

## Abstract

Social cognition is fundamentally interpersonal: individuals' behavior and dispositions critically affect their interaction partners' information processing. However, cognitive neuroscience studies, partially because of methodological constraints, have remained largely “perceiver-centric”: focusing on the abilities, motivations, and goals of social perceivers while largely ignoring interpersonal effects. Here, we address this knowledge gap by examining the neural bases of perceiving emotionally expressive and inexpressive social “targets.” Sixteen perceivers were scanned using fMRI while they watched targets discussing emotional autobiographical events. Perceivers continuously rated each target's emotional state or eye-gaze direction. The effects of targets' emotional expressivity on perceiver's brain activity depended on task set: when perceivers explicitly attended to targets' emotions, expressivity predicted activity in neural structures—including medial prefrontal and posterior cingulate cortex—associated with drawing inferences about mental states. When perceivers instead attended to targets' eye-gaze, target expressivity predicted activity in regions—including somatosensory cortex, fusiform gyrus, and motor cortex—associated with monitoring sensorimotor states and biological motion. These findings suggest that expressive targets affect information processing in manner that depends on perceivers' goals. More broadly, these data provide an early step toward understanding the neural bases of interpersonal social cognition.

## Introduction

Social life requires constant attention to and understanding of others' thoughts and feelings; as such, it is unsurprising that research has increasingly focused on the neural bases of these abilities (Decety, 2011; Zaki and Ochsner, 2012). The vast majority of this work has centered around the cognitive and neural processes engaged by perceivers (individuals focusing on another person's internal states) when they encounter social targets (individuals who are the focus of perceivers' attention). However, social cognition is fundamentally interpersonal, and social cognitive outcomes (such as interpersonal accuracy and rapport) depend just as deeply on targets' behaviors as they do on perceivers' skills and motives (Zaki and Ochsner, 2011).

For example, targets vary in their levels of emotional expressivity (i.e., the extent to which their behavior reflects their internal states). Expressivity can be measured either as a trait (e.g., through self-report questionnaires; see Gross and John, 1997) or as a state (e.g., by coding single episodes of behaviors such as emotional facial expressions; see Gross and Levenson, 1993). Trait and state measures of expressivity are moderately correlated, such that individuals who report themselves to be expressive also produce more clear and intense non-verbal emotional cues in experimental contexts (Gross and John, 1997; Gross et al., 2000; Zaki et al., 2009). Perhaps more importantly, expressivity measured as either a trait or a state predicts social outcomes. For example, targets high in trait expressivity are interpersonally “readable,” in that perceivers can accurately assess those targets' internal states (Snodgrass et al., 1998; Zaki et al., 2008; Zaki and Ochsner, 2011). State expressivity similarly predicts interpersonal accuracy (Zaki et al., 2009) and rapport (Butler et al., 2003).

How do targets' expressive traits and states exert their effects on interpersonal outcomes? Intuitively, we might expect that target attributes “get into the heads” of perceivers and affect their processing of social information. However, such an effect could reflect multiple mechanisms, because perceivers' responses to social cues depend heavily on the goals and cognitive resources they have on hand.

When given unconstrained cognitive resources (Gilbert et al., 1989; Epley and Waytz, 2009) and motivation to understand targets (Kunda, 1990), perceivers tend to draw explicit inferences about internal states based on targets' behavior and the context in which that behavior is embedded. Such “top down” social information processing is reliably accompanied by activity in a system of brain regions including the medial prefrontal cortex (MPFC), posterior cingulate cortex (PCC), precuneus, and temporoparietal junction (Fletcher et al., 1995; Gallagher et al., 2000; Mitchell et al., 2002; Ochsner et al., 2004; Saxe and Powell, 2006). Critically, inferential processing in this system is dependent on attention to targets' states (de Lange et al., 2008; Spunt et al., 2010; Spunt and Lieberman, in press).

However, perceivers do not always devote their full attention to understanding targets' thoughts and feelings; they are often distracted, otherwise occupied, or unmotivated to do so. Although this prevents perceivers from engaging in “top down” inferences, it nonetheless leaves room for a number of “bottom up” information processing mechanisms that draw on a system of brain regions almost wholly distinct from those accompanying explicit social inference (Whalen et al., 1998). For example, perceivers detect faces in their environment—a process drawing on the fusiform face area (FFA; see Kanwisher et al., 1997)—and vicariously share social targets' sensorimotor or visceral states—a process drawing on motor and somatosensory cortex (Rizzolatti and Craighero, 2004; Keysers et al., 2010)—even in the absence of explicit attention to targets' states (Vuilleumier et al., 2001; Winston et al., 2003; Chong et al., 2008; Spunt and Lieberman, in press).

Differences between the characteristics and neural underpinnings of top down and bottom up social processing suggest that target expressivity might affect perceivers' information processing, but in a manner that critically depends on task set. Specifically, when perceivers are directly attending to targets' internal states (e.g., emotions), expressive targets might provide a stronger “signal” on which to base top down social inferences, and increase perceivers' brain activity in regions associated with such inferences. By contrast, when perceivers are not explicitly attending to targets' states, expressive targets could nonetheless produce more salient social cues (e.g., more intense emotional facial expressions), which perceivers could evaluate using bottom up processes instantiated in a separate set of neural structures associated with perceiving faces or sensorimotor states.

The current study sought to test these possibilities. We presented perceivers with videos of social targets who varied in their levels of emotional expressivity, both as assessed through trait measures and through state ratings of their expressivity on a video-by-video basis. As such, trait and state expressivity provided “naturalistic” variance in the intensity of social cues produced spontaneously by social targets experiencing real emotions, as opposed to pictures of posed expressions whose intensity is manipulated by experimenters (Zaki and Ochsner, 2009). Perceivers viewed these targets in one of two conditions (1) while explicitly attending to targets' emotions, and (2) while attending to eye-gaze, a more low level feature of target behavior that is uncorrelated with the affect experienced or expressed by targets. This allowed us to directly test the prediction that target expressivity would modulate perceiver brain activity in a task-dependent manner.

More broadly, this study took an explicitly interpersonal tack toward the neural bases of social cognition. In part because of the highly intrapersonal nature of scanner environments, extant neuroimaging research has been almost entirely “perceiver-centric”: focusing on perceivers' skills, task sets, and motivations as determinants of judgment and predictors of neural activity. However, both intuition and behavioral research clearly support a more nuanced view of social information processing, in which perceivers' abilities and motivations interact with targets' behaviors and dispositions to produce interpersonal outcomes (Zayas et al., 2002; Zaki et al., 2008; Zaki and Ochsner, 2011). By directly examining such interactions at the level of the brain, the current study sought to provide early steps toward more deeply characterizing these “interactionist” (Mischel and Shoda, 1995) features of social cognition.

## Methods

### Stimuli

More detailed descriptions of the methods used here are available elsewhere (Zaki et al., 2008, 2009). In a stimulus collection phase of the study, targets (*N* = 14, 7 female, mean age = 26.5) were videotaped while talking about affective autobiographical memories (e.g., proposing marriage or the death of a loved one). Eighteen videos from 11 social targets were chosen for the final stimulus set, on the basis of their self-rated emotional intensity, and in order to balance the number of videos of each valence and target gender. The mean video length was 125 s (range: 72–177 s).

We examined target expressivity in two ways. First, trait expressivity was assessed through targets' responses to the Berkeley Expressivity Questionnaire (BEQ; see Gross and John, 1997; Gross et al., 2000). This measure captures targets' self-concept of how expressive they are (sample item: “when I'm happy, my feelings show”), and produced significant variance in our sample (mean BEQ score = 4.90, range = 3.69–6.47, SD = 1.02). In order to code “state” expressivity in each video, we used a behavioral coding system developed by Gross and Levenson (1993), which uses rules developed by Ekman and Friesen (1975/2003) to assess facial signs of emotion. We focused on the coding system's category: “affective intensity,” because it provides a single global measure of the strength of targets' non-verbal emotional displays (see Zaki et al., 2009 for more details). Two independent coders trained in the use of this system rated the average emotional intensity of each video, producing reliable ratings (Cronbach's alpha: 0.85; mean intensity score = 2.21, range = 1.17–4.02, SD = 0.61). As discussed elsewhere (Zaki et al., 2009) and found by others (Gross and John, 1997), targets' self-perceived trait expressivity as measured by the BEQ was correlated with the intensity of their non-verbal expressive behavior on a video by video basis, as assessed by independent raters (*r* = 0.28, *p* < 0.005).

### Protocol

Perceivers (*n* = 16, 11 female, mean age = 19.10, SD = 1.72) were scanned using fMRI while they watched all 18 target videos. While watching six of these videos, perceivers continuously inferred how positive or negative they believed targets felt at each moment; this will be referred to as the *emotion rating* condition. Under this condition, videos appeared in the center of a black screen; a cue orienting perceivers toward their task (e.g., “how good or bad was this person feeling?”) was presented above the video, and a nine-point rating scale (anchored at 1 = “very negative” and 9 = “very positive”) was presented below the video. Perceivers were instructed to change their rating whenever they believed target's emotional state changed in a perceptible way. At the beginning of each video, the number 5 was presented in bold. Whenever perceivers pressed the left arrow key, the bolded number shifted to the left (i.e., 5 was unbolded and 4 was bolded). When perceivers pressed the right arrow key, the bolded number shifted to the right. In this way, perceivers could monitor their ratings in the scanner.

While watching six other videos, perceivers were instructed to continuously rate how far to the left or right the targets' eye-gaze was directed; this will be referred to as the *eye-gaze rating* condition. The protocol for this condition was identical to the emotion rating condition, except that the task cue (“where is this person's eye gaze directed”) and Likert scale (1 = “far left,” 9 = “far right”) oriented perceivers toward the target's eye gaze. This task allowed us to examine brain activity evoked by perceivers' attending to targets, but not explicitly focusing on targets' internal states^1^.

Perceivers viewed videos under emotion rating and eye gaze rating in a pseudorandomized order, designed to ensure that (1) equal numbers of positive and negative videos were viewed by each perceiver under eye-gaze and emotion rating conditions, (2) equal numbers of videos featuring male and female targets were viewed by each perceiver under eye-gaze and emotion rating conditions, (3) no more than two consecutive videos were viewed under the same task (eye gaze or emotion rating), and (4) a roughly equal number of perceivers viewed each video under each task condition (e.g., a given video would be viewed by eight perceivers under the eye gaze condition, and by eight perceivers under the emotion rating condition). Finally, six additional videos were viewed under another condition not discussed here (see Zaki et al. (2012) for details about this condition).

### Imaging data acquisition

Images were acquired using a 1.5 Tesla GE Twin Speed MRI scanner equipped to acquire gradient-echo, echoplanar T2^*^-weighted images (EPI) with blood oxygenation level dependent (BOLD) contrast. Each volume comprised 26 axial slices of 4.5 mm thickness and a 3.5 × 3.5 mm in-plane resolution, aligned along the AC-PC axis. Volumes were acquired continuously every 2 s. Three functional runs were acquired from each subject. Because stimulus videos varied in length and were randomized across runs, the length of each run varied across subjects (range = 345–406 TRs). Each run began with five “dummy” volumes, which were discarded from further analyses. At the end of the scanning session, a T-1 weighted structural image was acquired for each subject.

### Neuroimaging analyses

Images were preprocessed and analyzed using SPM2 (Wellcome Department of Imaging Neuroscience, London, UK), and using custom code in Matlab 7.1 (The Mathworks, Matick, MA). All functional volumes from each run were realigned to the first volume of that run, spatially normalized to the standard MNI-152 template, and smoothed using a Gaussian kernel with a full width half maximum (FWHM) of 6 mm. Mean intensity of all volumes from each run were centered at a mean value of 100, trimmed to remove volumes with intensity levels more than three standard deviations from the run mean, and detrended by removing the line of best fit. After this processing, all three runs were concatenated into one consecutive timeseries for the regression analysis.

After preprocessing, we employed three analytic approaches using the general linear model. Across all three approaches, videos were modeled as blocks, in which the onset and duration of each video was convolved with a hemodynamic function. Our first analytic approach employed main effect contrasts to compare brain activity during the *emotion rating* and *eye-gaze rating* conditions; this served primary as a manipulation check, ensuring that attention to targets' emotion or to eye gaze preferentially engaged regions involved in making attributions about mental states and assessing low-level features of dynamic social stimuli (e.g., biological motion), respectively.

The second analytic approach directly addressed our primary hypotheses. Here, we used parametric analyses used to isolate perceiver neural structures in which activity varied as a function of target trait and state expressivity. In separate analyses, (1) targets' BEQ scores and (2) the intensity of emotional cues in each video were used as parametric modulators, providing regression weights for each video block. Using this method, we searched for clusters of activity that tracked—within perceivers—with the expressivity of targets they were watching; that is, regions that were more engaged when perceivers viewed a relatively expressive target, and less engaged when they viewed a relatively inexpressive target. These analyses were performed separately for the *emotion rating* and *eye-gaze rating* conditions.

Finally, to more directly assess the task dependency of expressivity related effects, we included two analyses aimed at isolating differences and similarities across eye-gaze and emotion monitoring. To examine differences across tasks, we computed a direct, whole brain analysis contrasting BOLD signal related to target expressivity (assessed at both state and trait levels) during emotion rating vs. eye gaze rating, and visa versa. This allowed us to directly assess an expressivity by task interaction in predicting perceivers' brain activity. To examine similarities across tasks, we computed a conjunction including maps reflecting expressivity-related activity in the eye-gaze rating and emotion-rating conditions, using the minimum statistic approach (Nichols et al., 2005). This analysis identifies clusters that were significantly engaged at our threshold in not one, but both conditions. Both of these analyses were performed separately for state and trait expressivity.

All analyses were thresholded at *p* < 0.005, with an extent threshold of *k* = 30. This cluster size was selected to correspond with a corrected threshold of *p* < 0.05, based on Monte Carlo simulations implemented in Matlab (Slotnick et al., 2003).

## Results

### Behavioral data

To assess participants' engagement during the session, we measured response rates: the number of times that perceivers changed their ratings per minute in each of the conditions. Individuals made significantly more ratings during the eye-gaze rating (mean = 14.11 ratings/minute) condition than during emotion rating (mean = 9.83 ratings/minute) condition, *t*(15) = 3.17, *p* < 0.01. Across both conditions, participants on average made ratings at least one rating per each 6.1 s, suggesting that they were engaged in both tasks.

### Neuroimaging data

#### Manipulation checks: neural bases of emotion rating vs. eye-gaze rating

**Figure 1:**
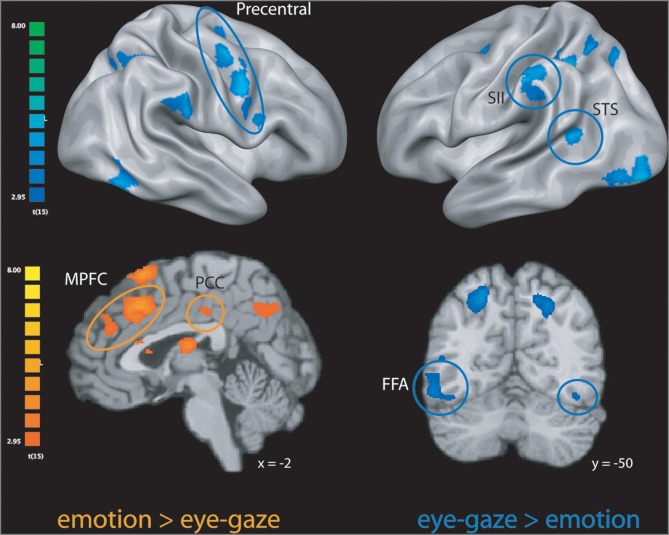
**Clusters more engaged during *emotion rating* than during *eye-gaze rating* (in orange); clusters more engaged during *eye-gaze rating* than during *emotion rating* (in blue).** STS, superior temporal sulcus; FFA, fusiform face area; MPFC, medial prefrontal cortex; PCC, posterior cingulate cortex. All clusters exceed a significance thresholded of *p* < 0.005, uncorrected, with an extent threshold of at least 30 voxels, corresponding with a threshold of *p* < 0.05, corrected as computed using Monte Carlo simulations.

We first explored neural activity distinctly engaged when perceivers explicitly attended to targets' internal states (*emotion rating*) and when they attended to lower-level features of target behavior (*eye-gaze rating*). As predicted, emotion rating—when compared to the eye-gaze monitoring—engaged brain regions classically associated with drawing inferences about mental states, including large clusters in MPFC, PCC, and precuneus (see Figure 1 and Table 1), as well as a number of clusters in left ventral and dorsal prefrontal cortex potentially related to the cognitive components necessary to making high-level emotional appraisals (Mitchell, 2009).

**Table 1:** **Main effects of condition**.

**Region**	**Coordinates**			**T-score**	**Volume (vox)**
	***x***	***y***	***z***		
**EMOTION RATING > EYE-GAZE MONITORING**					
ACC/MPFC	−2	24	42	6.2	1255
ACC/MPFC	−6	18	12	5.25	485
MPFC	−8	42	28	4.24	148
Middle Frontal Gyrus	−26	44	34	3.94	147
Middle Frontal Gyrus	−46	8	46	4.63	80
Middle Frontal Gyrus	−34	26	46	4.14	122
Inferior Frontal Gyrus	−46	40	−6	5.57	64
Inferior Frontal Gyrus	−44	24	−6	4.18	72
Dorsolateral Prefrontal Cortex	−46	26	26	4.48	45
Frontal Operculum	−56	14	10	5.18	232
Caudate	12	8	10	4.1	58
Precuneus/PCC	0	−22	40	4.85	161
Precuneus/PCC	−2	−64	40	3.66	175
Fusiform Gyrus	24	−76	−10	3.83	197
Striate Visual Cortex	−16	−70	−10	5.47	355
Cuneus	2	−84	22	3.9	116
**EYE-GAZE MONITORING > EMOTION RATING**					
Premotor Cortex	−26	−6	46	6.15	577
Premotor Cortex	−58	2	36	3.81	25
Premotor Cortex	54	0	36	5.65	1316
Supplementary Motor Area	8	−4	62	3.37	37
SII	64	−24	24	5.65	363
Superior Parietal Lobe	20	−62	56	5.59	770
Intraparietal Sulcus	−32	−42	48	5.48	1219
Fusiform Gyrus/STS	54	−58	−10	5.10	729
Fusiform Gyrus	−44	−48	−14	4.67	144
Extrastriate Visual Cortex	−42	−80	−6	6.67	661
STS	−51	−52	10	4.26	54

Note: Coordinates are in MNI space. ACC, Anterior Cingulate Cortex; MPFC, Medial Prefrontal Cortex; PCC, Posterior Cingulate Cortex; SII, Secondary Sensory Cortex.

The opposite comparison revealed that monitoring and rating targets' eye-gaze, as opposed to their emotional states, recruited a network of brain regions involved in monitoring motor intentions, somatosensory states, and biological motion, including bilateral pre-motor cortex, pre- and post-central gyrus, superior temporal sulcus, and SII, as well as bilateral inferotemporal cortex extending into the fusiform gyrus (see Figure 1 and Table 1).

#### Expressivity during emotion rating

**Figure 2:**
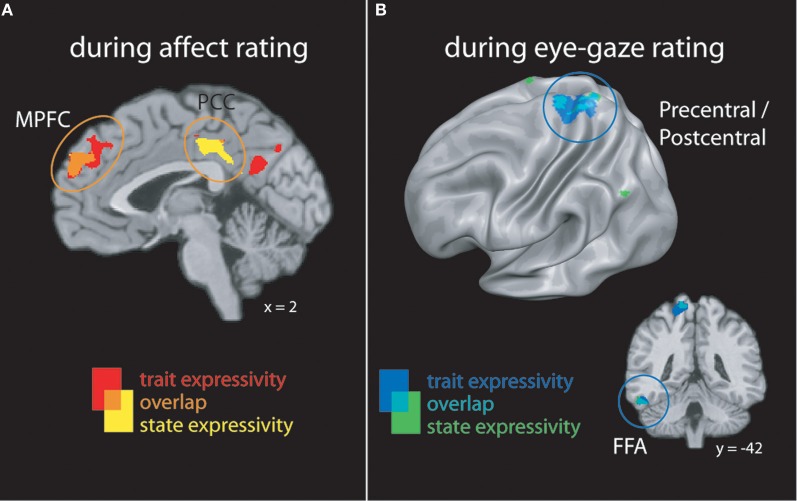
**(A)** Clusters whose activity tracked with targets' trait or state expressivity during *emotion rating*. **(B)** Clusters whose activity tracked with targets' trait or state expressivity during *eye-gaze rating*. FFA, fusiform face area; MPFC, medial prefrontal cortex; PCC, posterior cingulate cortex.

When perceivers were tasked with explicitly rating affective states, both targets' trait and video-by-video expressive behaviors were associated with increasing activity brain regions involved in mental state inference, including dorsal and rostral MPFC, PCC, and lateral temporal cortex (see Figure 2A and Table 2).

**Table 2:** **Modulation of brain activity by target expressivity**.

**Region**	**Coordinates**			**T-score**	**Volume (vox)**
	***x***	***y***	***z***		
**DURING EMOTION RATING (TRAIT EXPRESSIVITY)**					
MPFC	0	60	28	4.34	560
MPFC	−10	38	62	4.91	118
PCC/Precuneus	−4	−59	28	4.41	179
Superior Frontal Gyrus	−38	10	44	4.12	94
Middle Temporal Gyrus	68	−24	−18	4.82	177
Middle Temporal Gyrus	−60	−34	−22	3.59	106
**DURING EMOTION RATING (STATE EXPRESSIVITY)**					
MPFC	18	57	28	4.73	541
MPFC	4	50	0	4.58	48
MPFC/ACC	2	36	42	4.13	31
PCC	−2	−32	40	4.14	296
Inferior Temporal Gyrus	54	−30	−26	4.57	141
Posterior Parietal Lobe	−48	−62	38	4.41	74
Posterior Parietal Lobe	34	−78	52	4.28	67
Superior Frontal Gyrus	42	16	44	5.54	217
Precentral Gyrus	−42	−18	36	5.31	106
Inferior Temporal Gyrus	−70	−24	−18	4.78	60
**DURING EYE-GAZE RATING (TRAIT EXPRESSIVITY)**					
Premotor Cortex	18	−8	72	4.02	81
Precentral Gyrus	−19	−28	68	4.95	302
Precentral Gyrus	−36	−20	36	4.70	31
Fusiform Gyrus	−49	−42	−22	4.98	50
Middle Frontal Gyrus	50	30	32	4.99	57
Extrastriate Visual Cortex	−14	−82	30	3.68	71
Posterior Occipital Lobe	−24	−100	−10	4.34	103
Angular Gyrus	−52	−68	44	4.08	50
**DURING EYE-GAZE RATING (STATE EXPRESSIVITY)**					
Premotor Cortex	20	6	64	4.07	59
Precentral Gyrus	18	−8	72	3.83	45
Pre/Postcentral Gyrus	−20	−32	78	4.30	160
Fusiform Gyrus	−52	−40	−24	3.84	55
Inferior Frontal Gyrus	−24	20	−32	3.90	43
Caudate	−14	0	−8	4.08	32
Posterior Parietal Lobe	−50	−60	42	3.72	31

Note: Coordinates are in MNI space. ACC, Anterior Cingulate Cortex; MPFC, Medial Prefrontal Cortex; PCC, Posterior Cingulate Cortex.

#### Eye-gaze rating

When perceivers were instructed to monitor and rate eye-gaze direction—a more “low level” feature of target behavior—targets' trait and state expressivity tracked parametrically with activity in a set of brain regions involved in monitoring sensorimotor states and perceiving faces, including pre- and post-central gyri and left inferotemporal cortex spanning the fusiform gyrus (See Figure 2B and Table 2).

#### Direct comparisons across conditions

In order to compare expressivity related activity across eye gaze and emotion rating conditions, we computed a contrast isolating brain activity that was more responsive to target trait and state expressivity in the emotion rating, as compared to eye-gazing condition, and visa-versa. Broadly, the results of this analysis were consistent with the single-condition analyses. Critically, MPFC and several temporal lobe clusters originally identified as tracking expressivity during emotion rating were also significantly more responsive to target expressivity during emotion rating, as compared to eye gaze rating, regardless of whether expressivity was operationalized as a state or trait. The reverse analysis—isolating brain regions that respond to target expressivity more during eye-gaze rating than emotion rating—similarly identified regions found in the single-condition analysis, including the precentral gyrus and extrastriate visual cortex (Table 3).

**Table 3:** **Direct comparisons of expressivity related effects across conditions**.

**Region**	**Coordinates**			**T-score**	**Volume (vox)**
	***x***	***y***	***z***		
**EMOTION RATING > EYE GAZE RATING (TRAIT EXPRESSIVITY)**					
MPFC	−2	60	30	4.43	409
MPFC	0	36	48	3.49	39
Superior Frontal Gyrus	−40	18	46	3.91	139
Superior Temporal Gyrus	−60	−38	16	4.71	118
MTG/ATL	−62	−14	−16	4.06	154
Precentral/Postcentral Gyri	48	−16	36	4.38	62
**EMOTION RATING > EYE GAZE RATING (STATE EXPRESSIVITY)**					
MPFC	−6	58	30	4.23	229
Middle Frontal Gyrus	−42	10	44	4.8	171
Anterior Temporal Lobe	58	0	−36	4	41
Middle Temporal Gyrus	52	−4	−12	3.74	44
Inferior Temporal Gyrus	−52	−24	−26	3.89	75
Precentral Gyrus	44	−18	36	3.86	55
**EYE GAZE RATING > EMOTION RATING (TRAIT EXPRESSIVITY)**					
Precentral Gyrus	28	−22	64	6.17	36
Ventral Striatum	4	2	−2	4.56	117
Fusiform Gyrus	36	−78	−2	3.8	30
**DURING EYE-GAZE RATING (STATE EXPRESSIVITY)**					
Cerebellum	−2	−54	−42	4.78	148
Fusiform Gyrus	36	−76	2	4.6	123
Medial Occipital Lobe	16	−88	26	3.65	37

Note: Coordinates are in MNI space. MPFC, Medial Prefrontal Cortex; MTG, Middle Temporal Gyrus; ATL, Anterior Temporal Lobe.

That said, this direct contrast did not entirely reproduce the findings of our single-condition analyses. Specifically, whereas activity in PCC was found to track expressivity during emotion rating, but not eye-gaze rating, this region was not significantly *more* responsive to expressivity under one condition, as compared to the other. Similarly, whereas the fusiform gyrus (corresponding to the so-called “face area”) was responsive to target expressivity under the eye-gaze rating, but not emotion rating condition, this region was not significantly more responsive to target expressivity under eye-gaze rating, as compared to emotion rating, under a direct comparison.

Finally, to isolate any regions whose activity commonly tracked expressivity across both tasks, we computed a conjunction analysis between both activation maps from our original parametric analysis (corresponding to expressivity-related activity under each condition), separately for trait and state expressivity. This analysis revealed very little common activation across tasks. In fact, only one cluster survived either conjunction: during both eye-gaze and emotion-rating, targets' trait expressivity predicted activity in the postcentral gyrus (xyz coordinates: −24, −40, 60, *t* = 3.52, *k* = 41 voxels).

## Discussion

Perceivers do not employ social cognitive processes in a vacuum. On the contrary, social cognition is deeply interpersonal, and social psychologists have long studied the way that people's traits and states affect the cognitions, affect, and physiology of their interaction partners (Snodgrass et al., 1998; Butler et al., 2003). However, methodological constraints have often prevented neuroimaging researchers from studying the way that one person's traits or behaviors “get into perceivers' heads,” and influence cognitive and neural processes they engage (although newer methods are increasingly circumventing these issues; see, for example Wilms et al., 2010). Further, little work has examined how the intensity of social stimuli (including social targets' expressivity) interacts with perceivers' goals to affect information processing.

The current study addressed both of these gaps in knowledge. Perceivers watching videos of naturally expressive, as opposed to inexpressive, social targets demonstrated increased engagement of several brain regions, regardless of whether expressivity was measured as a trait (through self-report questionnaires) or as a state (through coding of targets' video-by-video emotional behavior). However, the patterns of neural activity associated with target expressivity depended on perceivers' information processing goals. If perceivers were actively evaluating targets' emotions—a task drawing on areas involved in drawing top-down inferences about internal states, such as the MPFC and PCC—then expressivity modulated activity in these areas. If, instead, perceivers were attending to targets' dynamic shifts in eye-gaze, then target expressivity correlated with activity in a wholly separate set of brain regions, including areas associated with processing faces and biological movement, as well as cortical regions involved in simulating targets' sensorimotor states.

The positive relationship between target expressivity and perceivers' engagement of key neural associated with social cognition suggests that more expressive targets somehow “amplify” processing related to decoding others' internal states. This amplification could reflect at least two separable effects. First, expressive targets could produce clearer (i.e., more “readable”) social and affective signal, which in turn allow perceivers to mentalize more effectively. Second, expressive targets may produce the types of salient signals (e.g., intense facial expressions) that spontaneously draw perceivers' attention, and thus cause those perceivers to engage more deeply in subsequent mentalizing and processing of sensorimotor social cues. Further research should examine the extent to which expressivity-driven amplification reflects each or both of these effects.

### Implications and future directions

#### Expressivity as a window into social cognitive “processing streams”

Perhaps the most striking finding of the current study is that perceivers' task set strongly determined the neural correlates of target expressivity, and that expressivity effects recapitulated the main effect differences between top-down and bottom-up social information processing. When perceivers attended to targets' affect they preferentially drew on brain regions involved in drawing explicit inferences about targets, whereas attention to target eye gaze engaged regions involved in more automatically processing faces, biological motion, and sensorimotor cues.

Critically, this dissociation was broadly paralleled by the effects of target expressivity, which drove activity in regions associated with explicit mental state attribution or bottom up processing of social stimuli when perceivers attended to targets' emotions or eye gaze, respectively. A direct comparison across tasks revealed that activity in some of these key regions was significantly more related to target expressivity under bottom-up or top-down social cognitive processing goals. MPFC and several lateral temporal regions were more strongly engaged by target expressivity during emotion rating, as compared to eye gaze rating, whereas the precentral gyrus and extrastriate visual cortex demonstrated the opposite pattern. Other regions—such as the PCC and fusiform gyrus (adjacent to the so-called “face area”) tracked expressivity in only one of these conditions, but did not significantly differentiate between conditions. These regions may be somewhat engaged across both conditions, but fail to meet a significance threshold under one condition. Consistent with this idea, a conjunction analysis revealed that almost no clusters of brain activity significantly tracked target expressivity across both conditions. Together, these data suggest that the effects of target expressivity on perceivers' brain activity strongly—but not entirely—depends on perceivers' information processing goals.

This finding lends converging support to the idea of separable social cognitive “processing streams” (Zaki and Ochsner, 2012; Zaki, under revision). The first, centered in midline and lateral temporal cortex, is likely involved in perceivers' ability to simulate targets' experiences (Buckner and Carroll, 2007; Spreng et al., 2009), and likely requires perceivers to explicitly attend to targets (de Lange et al., 2008; Spunt and Lieberman, in press). The second, distributed among regions involved in processing low-level social visual cues (e.g., faces and biological movement) and engaging somatosensory states expressed by targets, is engaged in a task-independent fashion (Chong et al., 2008), and deployed whenever the environment contains relevant social cues (Spunt and Lieberman, in press). In fact, this second processing stream is sometimes most engaged when perceivers do *not* explicitly attend to targets' internal states (Lieberman et al., 2007). The dissociation between these social cognitive processing streams has now been established across a number of studies (Brass et al., 2007; Gobbini et al., 2007; Wheatley et al., 2007; Spunt and Lieberman, in press), and meta-analyses (Van Overwalle, 2009; Van Overwalle and Baetens, 2009). Here, we extend this finding by demonstrating that not only are top down and bottom up processing streams dissociable, but that identical variance in the intensity of social cues (here instantiated through target expressivity) will affect one of these processing stream or the other, independently, as a function of perceivers' current goals and cognitive resources.

The relationship between target expressivity and perceiver goals in predicting brain activity further bolsters an “interactionist” (Mischel and Shoda, 1995) model of social cognition as a fundamentally interpersonal phenomenon: depending on the states and traits of not one person, but of both targets and perceivers. This framework has been used to fruitfully capture variance in social judgments and behaviors (Snodgrass et al., 1998; Zayas et al., 2002; Zaki et al., 2008, 2009; Zaki and Ochsner, 2011). Here we extend this approach to modeling brain activity. Importantly, the paradigm used here was not “interactive,” in that it did not include online interactions between—or record brain activity from—both targets and perceivers (Schilbach et al., 2006, 2011; Schippers and Keysers, 2011). However, interactionist models of social cognition like the one supported here dovetail nicely with interactive paradigms to support more holistic models of social cognition and interaction (Zaki and Ochsner, 2009; Schilbach et al., 2012).

#### Stimulus intensity and naturalistic social cues

Although prior work has almost never focused on the neural bases of processing information about expressive vs. inexpressive social targets, a few prior studies have examined the effects of affective stimulus intensity on brain activity, in the domains of odor (Small et al., 2003), words (Cunningham et al., 2007), and faces (Winston et al., 2003). In all of these cases, stimulus intensity predicted amygdala activity, whereas in the current study it did not. One possibility is that our design—which employed a relatively small number of stimuli and a parametric analysis—may have been underpowered to detect effects in the amygdala. A second possibility is that a lack of amygdala activity in our task could reflect differences between the types of cues employed in previous studies of emotion perception and more “naturalistic” cues produced by real social targets (Zaki and Ochsner, 2009). Even during the most intense emotional experiences (e.g., after winning an Olympic gold medal) targets typically produce complex, nuanced facial expressions that differ fundamentally from the posed, canonical displays often used in research (Russell et al., 2003). Thus, while the amygdala is clearly important to forming fast and computationally efficient evaluations of many affective stimuli, its role in reacting to and interpreting the more subtle cues produced by social targets in many other situations may be more limited.

More broadly, our data connect with the literature on processing affective cues under different levels of attention. Specifically, prior work has demonstrated that affective stimuli engage several neural structures—including the amygdala and sensorimotor cortex—when perceivers do not attend to target affect (Spunt and Lieberman, 2012; Whalen et al., 1998; Winston et al., 2003), attend to low-level target features including eye gaze (Adams and Franklin, 2009), or draw inferences about targets based on non-verbal cues (Kuzmanovic et al., 2011). Although researchers have debated the extent to which neural responses to affective cues are truly automatic (Pessoa et al., 2002; Pessoa, 2005), the modulation of affect-related neural processing by, for instance, top down vs. bottom up processing goals is rapidly becoming an established feature of the neuroscientific literature. Here, we extend this insight to demonstrate that naturally occurring variance in target expressivity modulates neural activity in a manner broadly consistent with such task dependency.

#### Target expressivity as a buffer against social cognitive dysfunction

One especially interesting application of the current approach surrounds illnesses that involve social cognitive and behavioral dysfunctions. Such difficulties characterize a raft of psychiatric disorders, such as schizophrenia, borderline personality disorder, and social phobia. In almost all cases, social deficits in these conditions are studied using standardized social stimuli and paradigms. However, social deficits in these conditions could critically depend not only on the cognitive or affective characteristics of affected perceivers, but also on the dispositions and behaviors of the targets they encounter. Consider a condition heavily associated with social cognitive dysfunction: Autism Spectrum Disorders (ASD). Individuals with ASD perform poorly on social cognitive tasks such as mental state inference (Roeyers et al., 2001), a deficit that has been tied to attenuated activation of several brain regions including the MPFC and FFA (Schultz et al., 2000, 2003; Wang et al., 2007). However, perceivers with ASD perform as well as control participants at a social inference task when social cues are presented in a clear and structured manner (Ponnet et al., 2007). One intriguing possibility is that expressive targets may provide exactly these types of clear social cues, and perceivers with ASD may demonstrate more normative behavior and patterns of brain activity when observing expressive targets (Zaki and Ochsner, 2011). Such a finding would have implications for potential intervention approaches focused on teaching caretakers and peers of individuals with ASD to structure their social cues in a manner that drives social cognitive processing and performance in those individuals. Such an approach has the potential to expand ASD interventions to encompass both perceivers' and targets' roles in producing accurate and adaptive social cognition.

## Conclusions

The current study demonstrates that the neural bases of social inference are modulated by interpersonal factors. Social targets' trait expressivity affected perceivers' deployment of social cognitive processing, but in ways that depended on the task perceivers were performing. These data provide an early step toward using neuroimaging to unpack the processes involved in fundamentally interpersonal social cognition.

### Conflict of interest statement

The authors declare that the research was conducted in the absence of any commercial or financial relationships that could be construed as a potential conflict of interest.

## Footnotes

^1^Eye-gaze and eye-gaze direction are, at some level, social cues (Macrae et al., 2002; Mason et al., 2005), which, in this case, might pertain to emotions expressed by the individuals in the video, and attending to eye-gaze can engage some neural structures commonly associated with social inference (Calder et al., 2002). As such, comparing emotion rating with eye-gaze rating provided an especially conservative contrast that focused specifically on explicit attention to emotion, as opposed to incidental processing of social information (see “Discussion”) or attentional and motoric demands.
